# The Exceptional Oncogenicity of HTLV-1

**DOI:** 10.3389/fmicb.2017.01425

**Published:** 2017-08-02

**Authors:** Yutaka Tagaya, Robert C. Gallo

**Affiliations:** Division of Basic Science, Institute of Human Virology, University of Maryland School of Medicine Baltimore, MD, United States

**Keywords:** HTLV-1, retrovirus, oncogenicity, T-cell leukemia, oncoviruses

## Abstract

Human T-cell leukemia virus-1 (HTLV-1) is the first pathogenic human retrovirus identified in 1979 by the Gallo group. HTLV-1 causes fatal T-cell leukemia (adult T cell leukemia) and a progressive myelopahy (HTLV-1-associated myelopathy/ tropical spastic paraparesis, HAM/TSP) and other disorders. Since the discovery of HTLV-1, several other microorganisms are demonstrated to cause cancer in humans. In this article, we investigated the oncogenic capacity of HTLV-1, in comparison with those of other oncoviruses and one oncobacterium (Helicobacter pylori, H. Pylori) based on published literature. We conclude here that HTLV-1 is one of the most and may be the most carcinogenic among them and arguably one of the most potent of the known human carcinogens. This fact has not been noted before and is particularly important to justify why we need to study HTLV-1 as an important model of human viral oncogenesis.

At the time of discovery of HTLV-1 (Poiesz et al., 1980a,b; Hinuma et al., 1981), the concept that viruses could cause cancer (ATL, adult T-cell leukemia/lymphoma by HTLV-1) in humans was a far-fetched idea that the medical community eventually accepted, despite the earlier identification of Epstein-Barr virus in cultured lymphoblasts from Burkitt's lymphoma (Epstein et al., 1964). After 35 years, the concept of viral oncogenesis has become a textbook fact and several viruses have joined the group of human oncoviruses. In addition, there is now one oncogenic bacterium (Helicobacter pylori) and a strong suspicion of more. The list of these oncogenic microbes, their global burden, and the incidence of malignant disorders involving them are provided in Table 1 and summarized below.

**Table 1:** Summary of the statistics on malignancy development associated with oncogenic microorganisms.

**Infectious organism**	**Global burden (% in population)**	**Lifetime risk of malignancy development following infection**
H. Pylori	5.5	3%
HPV	5.2	0.29%
HCV + HBV	4.9	HCV, 1~3%/HBV, <1%
HHV-8	2~5	Minimally oncogenic by itself
EBV	>90	0.3~0.4%
HTLV-1	0.3% (~40% in central Australian aboriginals)	5~10%
MCV	0.06	??
AAV2	??	??

The mechanism by which a virus causes tumor differs from virus to virus. While some viruses seem to require co-factors, and/or work indirectly such as by micro-environmental alterations, others directly transform host cells. One thing in common is that there are not yet effective cures for viral malignancies in humans. We conducted a literature survey in order to compare the carcinogenic potency of known oncoviruses, and present the risk of malignancy development following the infection of each oncovirus.

## Epstein barr virus (EBV)

In developing countries, the prevalence of EBV infection reaches over 90% before the age of 20. But even in high-risk area (East Asia), the incidence of nasopharyngeal carcinoma in association with EBV is at best 0.05% or less. Numbers for endemic Burkitt's lymphoma is more imprecise but estimated as 3~4/100,000 among Ugandan children (Hjalgrim et al., 2007), one of the most endemic area of EBV-related Burkitt's lymphoma (BL). It is also of note that the EBV-mediated malignancy development generally requires a co-factor, especially malaria in the case of BL and dietary factors in nasopharyngeal carcinoma. Elsewhere, the role of EBV as an oncogenic agent remain unclear. Thus, EBV is an oncogenic virus but one with very low level of oncogenicity and appears to be only one factor in a multi-factorial cause.

## Human herpesvirus-8 (HHV-8)

Though it is a cause of Kaposi's sarcoma (HHV-8 is thus also known as the Kaposi Sarcoma-associated herpesvirus) and a more special and very atypical form of lymphoma in collaboration with HIV, HHV-8 is very minimally oncogenic in the absence of HIV. We also note that in most of primary effusion lymphoma cases associated with HHV-8, EBV is detected along with HHV-8, another support that HHV-8 is not potently oncogenic on its own (Bhutani et al., 2015).

## Human papilloma virus (HPV)

More than 100 HPVs exist, and at least 13 of them are associated with human cancers (zur Hausen, 2009). The high rise types 16 and 18 are particularly responsible for 60~70% of cervical cancer worldwide (Khan et al., 2005). More than 80~90% of sexually active men and women will be infected with at least one type of HPV at some point in life and nearly 50% of these infections are with a high risk HPV type (Hariri et al., 2011). In the US, these high-risk HPV causes 2~3% of all cancer cases among men and women (Jemal et al., 2013). This translates to 0.29% of the newly infected individuals with high-risk HPVs eventually developing HPV-related cancer. HPV infection can cause cervical, vaginal, and vulvar cancers, cancers of the penis or anus, as well as some head and neck cancers. Vaccines against HPV 16 and 18 have received approval in many countries.

## Hepatitis C virus (HCV)/hepatitis B virus (HBV)

[HCV] 85% of acute HCV infections will be unresolved, developing into chronic infection. Importantly, there is no evidence to support the conclusion that HCV is directly oncogenic, nonetheless it is clearly associated with hepatocellular carcinoma (HCC). That said, one study estimates that the HCC develops in 1~3% of HCV infected persons after 30 years and the mechanisms are obscure (Goodgame et al., 2003). A chronic infection with HBV establishes when infection occurs in infants and children.

[HBV] <10% of healthy adults over 19 years will develop chronic hepatitis following HBV infection. A study conducted in Taiwan, a hyperendemic area of chronic HBV, shows that lifetime risk of HCC with HBsAg seropositive is 6.96% among women, but is an amazing 27.4% among men with HBsAg seropositivity (Huang et al., 2011). In a study conducted in the US, the 5 year HCC cumulative incidences with HBV-associated cirrhosis was 10% (Fattovich et al., 2008). Considering that <10% of HBV infected healthy adults develop chronic infection, the percentages of HCC development by HBV infection should be much lower than 1%.

## Adeno-associated virus type 2 (AAV2)

This is the newest member to the family of oncogenic microorganisms (Nault et al., 2015). The oncogenesis of AAV2 is associated with HCC without cirrhosis (thus distinguishing then from those caused by hepatitis virus B or C). A clonal integration of AAV2 was found in 11 of 193 HCC cases in which AAV2 integrations occurred in cancer driver genes such as cyclin A2, E1, Telomerase reverse transcriptase etc. These insertions of AAV2 led to the over-expression of target genes. There is still very limited information available for the epidemiological facts about HCC caused by AAV2.

## Merkel cell polyomavirus (MCV)

MCV was discovered in 2008 (Feng et al., 2008) and causes a rare but aggressive neuroendocrine tumor (Merkel cell carcinoma, MCC) of the skin and its incidence seems rising. A 5 year mortality rate is as high as 46%. The annual incidence of MCC is 0.6 per 100,000 persons (0.06%) and about 1,600 new cases annually appear in the US. This rise in incidence may be partly because of the increased awareness and improved diagnostic methodology. The median age at diagnosis is >70 years. Only 4% of patients are diagnosed at 50 year or younger and it is extremely rare in children (Hughes et al., 2014). The oncogenic mechanism of MCV is still under investigation. No viral elements have been shown to directly cause cellular transformation. Nor does MCV seem to exploit cellular oncogenic mechanisms (p53, PTEN, Raf, Ras, etc.) (Lemos and Nghiem, 2007). Increase of MCC upon UV irradiation is reported, suggesting a potential involvement of defective DNA repair mechanism in the carcinogenesis (Lunder and Stern, 1998). In addition, a weakened immune function may be related to the occurrence of MCC. For example, the incidence of MCC is 5~10 fold higher in immune-compromised individuals with AIDS or solid organ transplant (Becker, 2010). Some anecdotal regression of MCC has been observed following improvement in immune function (Wooff et al., 2010). As with most other oncogenic viruses, the presence of MCC seems insufficient to induce MCC and additional cellular events together with the loss of immune-surveillance are postulated. In summary, there is still missing information to evaluate the oncogenic potential of MCV in comparison with other oncogenic viruses, however MCC caused by MCV may represent another case of aggressive viral oncogenesis.

## Human immunodeficiency virus-1 (HIV-1)

HIV is not generally listed with the oncogenic viruses by most virologists. However, in some reports it is analogous to HCV in that from an epidemiological view it is related to the frequency of developing cancer, notably non-Hodgkin lymphomas, Kaposi sarcoma, and cervical cancer that are known as AIDS-defining malignancies (reviewed in Levine, 1993). The mechanism(s) of cancer is indirect and relates to the microenvironment and perhaps to the diminished immune surveillance because the virus is rarely found in the tumor cells. The incidence of cancer development in HIV infected persons is about 40% (Levine, 1993) and usually accompanies co-infection with other oncoviruses.

## Human T-cell leukemia virus-1 (HTLV-1)

The oncogenic nature of HTLV-1 is solid; (1) Epidemiology studies (recently reviewed by Gessain and Cassar (Gessain and Cassar, 2012), (2) Clonal integration of the HTLV-1 in ATL cells (Hahn et al., 1983). This indicates that the virus was present at the level of the progenitor cell that gave rise to the leukemia. (3) Animal models that recapitulate human leukemia/lymphoma development by HTLV-1 or its genetic components (Tax-1, HBZ) of HTLV-1 (Hasegawa et al., 2006; Satou et al., 2011), (4) Immortalization of T cells by the Tax-1 gene of HTLV-1 (Grassmann et al., 1989), (5) Reproduction of the leukemia/lymphoma by the whole virus. The prevalence of ATL is 3–5% among infected persons and HTLV-1 infection is 0.1% as global average but the epidemiological data are still incomplete because those from highly populated areas such as China, India, the Maghreb, and East Africa are still unavailable. ATL is extremely difficult to treat, to the point that patients diagnosed with aggressive forms (acute and lymphomatous phases) were estimated to have <1 year of life left. However, recent years have seen promising developments of novel treatment modalities including arsenic trioxide (Bazarbachi et al., 2011), allogeneic stem cell transplant (Utsunomiya et al., 2001; Katsuya et al., 2015) and antibody therapy involving humanized anti-CCR4mAb (Ishida and Ueda, 2011; Katsuya et al., 2015). However, the establishment of a curative treatment for ATL probably needs more creative ideas.

## Helicobacter pylori (H. Pylori)

Helicobacter is a bacterium, and represents the first example of what may be emerging examples of bacterial oncogenesis. It has been somewhat controversial how infection with helocobacter pylori would increase or decrease various type of cancer in humans. A recent study in Japan (involving 1,526 patients) reported that gastric cancer develops in approximately 3% of H. pylori-infected patients, compared to none of the uninfected patients (Uemura et al., 2001). This number matches the development of Adult T cell Leukemia/lymphoma (ATL) in HTLV-1 infected individuals.

In summary, we note here that HTLV-1 is one of the most oncogenic entities known among human viruses and even among most known human carcinogens. The oncogenic mechanism of HTLV-1 is more direct compared with other entities and to date there is no evidence for any particular co-factor requirement. This striking characteristic of HTLV-1 seems overlooked because of its low prevalence in the U.S. and Europe. As discussed in an accompanying paper, we propose to revert the name of HTLV-1 back to the original, Human T-cell leukemia virus. This is also supported by a recent survey among 21 HTLV-1 experts from all over the world who belong to the HTLV-1 task-force of the Global Virus Network (GVN, http://gvn.org), a coalition of virologists on a global scale, as the result of the vote (16 vs. 5) supported the original name “leukemia”. Another vote conducted at the 18th International HTLV-1 meeting (Tokyo, 2017) again showed majority support (78 vs. 26) on the “leukemia” name. We believe that this most carcinogenic virus deserves the “leukemia” name.

## Author contributions

YT and RG conceptualized the idea, conducted literature search and investigation, and wrote the manuscript.

### Conflict of interest statement

The authors declare that the research was conducted in the absence of any commercial or financial relationships that could be construed as a potential conflict of interest.
